# Genomic and Biological Profile of a Novel Bacteriophage, Vibrio phage Virtus, Which Improves Survival of *Sparus aurata* Larvae Challenged with *Vibrio harveyi*

**DOI:** 10.3390/pathogens11060630

**Published:** 2022-05-30

**Authors:** Stavros Droubogiannis, Pantelis Katharios

**Affiliations:** 1 Institute of Marine Biology, Biotechnology & Aquaculture, Hellenic Centre for Marine Research, 71500 Heraklion, Greece; stavros.drou@gmail.com; 2 Department of Biology, School of Sciences and Engineering, University of Crete, 71500 Heraklion, Greece

**Keywords:** *Vibrio harveyi*, phage therapy, aquaculture, gilthead seabream

## Abstract

Due to the emergence of multidrug-resistant bacteria, commonly known as “superbugs”, phage therapy for the control of bacterial diseases rose in popularity. In this context, the use of phages for the management of many important bacterial diseases in the aquaculture environment is auspicious. *Vibrio harveyi,* a well-known and serious bacterial pathogen, is responsible for many disease outbreaks in aquaculture, resulting in huge economic and production losses. We isolated and fully characterized a novel bacteriophage, Vibrio phage Virtus, infecting *V. harveyi* strain VH2. Vibrio phage Virtus can infect a wide spectrum of *Vibrio* spp., including strains of *V. harveyi*, *V. owensii*, *V. campbellii*, *V. parahaemolyticus*, and *V. mediterranei*. It has a latent period of 40 min with an unusually high burst size of 3200 PFU/cell. Vibrio phage Virtus has a double-stranded DNA of 82,960 base pairs with 127 predicted open reading frames (ORFs). No virulence, antibiotic resistance, or integrase-encoding genes were detected. In vivo phage therapy trials in gilthead seabream, *Sparus aurata*, larvae demonstrated that Vibrio phage Virtus was able to significantly improve the survival of larvae for five days at a multiplicity of infection (MOI) of 10, which suggests that it can be an excellent candidate for phage therapy.

## 1. Introduction

*Vibrio harveyi* belongs to the *Vibrionaceae* family and is an opportunistic, serious pathogen responsible for many disease outbreaks in marine animals worldwide. It is established as the main cause of gastroenteritis and vibriosis in various fish, crustacean, and molluscan species [1,2]. *V. harveyi* is ubiquitous and usually grows in temperatures above 18 °C, as its optimal temperature is 25 °C [3]. Climate change and the overall rise of the water temperature in the oceans, along with the intensification of aquaculture, favor the increase in vibrios, and hence the vibriosis incidents have increased alarmingly [3,4,5,6,7]. To date, the management of *Vibrio* infections has relied mostly on antibiotics such as oxytetracycline, flumequine, and ampicillin. [8]. However, the extensive use of such treatments is associated with the development of multidrug-resistant bacteria, affecting not only the management of the diseases in aquaculture but also humans since antimicrobial resistance (AMR) can be transmitted from livestock to humans [9]. Therefore, a new strategy for tackling the problems related to antibiotics in aquaculture is urgently required. Phage therapy, the use of phages as biocontrol agents, is considered a promising alternative [10,11]. The ease with which phages may be isolated, their abundance and host specificity, along with the high cost and effort required for the development of novel antimicrobial agents, have shifted the attention of scientific community to phages. There is an increasing number of studies regarding the application of phage therapy in aquaculture, yielding promising results [12,13,14]. However, phage therapy requires a thorough understanding of the bacteriophages being used, determining their genomic and biological characteristics. Here, we isolated and fully characterized a lytic bacteriophage, Vibrio phage Virtus, infecting the pathogenic, antibiotic-resistant *Vibrio harveyi* strain VH2 [15], and tested its efficacy in vitro against its host and in vivo using gilthead seabream, *Sparus aurata*, larvae.

## 2. Materials and Methods

### 2.1. Bacterial Strains

Twenty-six strains of *Vibrio harveyi, V. alginolyticus, V. owensii, V. anguillarum, V. campbellii, V. parahaemolyticus*, *V. campbellii*, and *V. rotiferianus* (Table 1) used in this study were obtained from the bacterial collection of the Laboratory of Aquaculture Microbiology, Institute of Marine Biology, Biotechnology and Aquaculture (IMBBC), Hellenic Center for Marine Research (HCMR) in Heraklion, Crete. The bacterial strains were previously identified either through their NCBI or ENA accession numbers for the type strains, biochemical test (BIOLOG GEN III), and PCR method (sequencing of 16s rRNA and toxR amplifications). Moreover, a strain of *Phaeobacter piscinae*, a kind offer of Prof. Lone Gram (DTU), was also included in the assays. All the bacterial strains were maintained in microbeads (MicroBank) at −80 °C and were grown in lysogeny broth (10 g/L tryptone, 5 g/L yeast extract, 10 g/L NaCl, 1 L deionized water, 0.75 g/L MgSO_4_, 1.5 g/L KCl, 0.73 g/L CaCl_2_) at 25 °C when used.

### 2.2. Isolation and Purification of Bacteriophages

Water samples were collected from a fish tank in the broodstock section of HCMR in Gouves, Heraklion, Crete. An overnight *Vibrio harveyi* VH2 culture in LB broth (2.5 mL) was added in 25 mL of concentrated LB and used for the enrichment of the water samples followed by incubation at 25 °C with a shaking speed of 80 rpm for 24 h. Subsequently, enrichments were centrifuged at 13,000 rpm for 3 min, and supernatants were filtered through a 0.22 µm sterile filter (GVS Life Sciences, Sanford, ME, USA). A total of 10 µL of each sample was spotted on bacterial lawns of the host strain. After an incubation for 24 h, the clearest plaques were collected and serially propagated against the host using the double agar layer method according to Clokie et al. as described in Misol et al. [16,17]. The same procedure was repeated five times. The purified phage was selected for further characterization and was designated Vibrio phage Virtus.

### 2.3. Transmission Electron Microscopy

A purified, high-titer (>10^11^ PFU mL^−1^) aliquot of the novel phage obtained following PEG centrifugation was used for electron microscopy observation. Negatively stained (4% *w/v* uranyl acetate, pH 7.2) samples were prepared at the Electron Microscopy Laboratory of the University of Crete as described previously by Misol et al. [17] and observed with a JEOL transmission electron microscope at 80 kV. Morphology and size of virions were obtained from digital micrographs using ImageJ (software version 1.53p) [18]. Measurements (*n* = 15) were obtained for capsid width, tail length, baseplate length, and baseplate width.

### 2.4. Lysogeny Test

To examine whether Vibrio phage Virtus can follow a lysogenic cycle, we developed phage-resistant host mutants following the method described in Thomas Denes et al. [19]. Briefly, a high titer of Vibrio phage Virtus was added in liquid cultures of host bacteria *V. harveyi* VH2, following a 24 h incubation. Samples were then taken from each culture and plated on LB agar. After incubation for 24 h, more than 20 colonies were isolated from each plate. Colonies that were resistant to phage and retained resistance through subsequent recultures, were selected and stored as phage-resistant mutants. We assume that if the phage was temperate, some of the phage resistant mutants would be lysogenized. Following that, prophage induction was conducted to 11 resistant mutants according to Jackel et al. [20] with minor modifications. Briefly, aliquots of overnight bacteria cultures at 10^7^ CFU mL^−1^ were mixed with top molten LB agar (0.75% agar) and poured on bottom LB agar. The Petri dishes were then placed in 10 cm to a UV lamp (6 W, 254 nm) for 5 s. The induction of any possible lysis was assessed the following day by examining plates for plaques.

### 2.5. Host Range Test

Host range analysis was conducted according to Misol et al. [17]. Fresh cultures of the bacterial strains used in this study (Table 1) inoculated LB broths at a concentration of approximately 10^7^ CFU mL^−1^. They were then mixed with top soft LB agar (0.75% agar) and poured on bottom LB/2 agar, which contained half of the tryptone and yeast content of LB agar. After the solidification of top agar, three spots of 10 μL of Vibrio phage Virtus were used for phage enumeration.

### 2.6. Efficiency of Plating (EOP)

Efficiency of plating (EOP) was performed according to Kutter et al. [21], as described in Misol et al. [17]. The phage was serially diluted to ≈10^−1^, 10^−2^, 10^−3^, 10^−4^, 10^−5^, 10^−6^, and 10^−7^ and spotted on the bacterial lawns of the 25 bacterial strains. Three spottings were used in order to assess phage titer after the agar plates were incubated at 25 °C for 24 h. The EOP was calculated as a percentage of the number of plaque-forming units formed on a bacterial strain against the number of plaque-forming units formed on the host *Vibrio harveyi* VH2. EOP more than 10 was categorized as high, EOP between 9.9 and 0.5 was considered medium, while EOP less than 0.5 was considered low.

### 2.7. Stability of Phage in Different Temperatures, pH Values, and Organic Solvents

The stability of the phage against different temperatures, pH values, organic solvents, and common disinfectants was assessed in order to determine phage versatility in therapy conditions. Phage aliquots of ≈10^11^ PFU mL^−1^ were exposed to different temperatures (25, 30, 35, 40, 45, 50, 55, and 65 °C) for 1 h before being rested at 25 °C for 10 min. Following serial dilutions, the aliquots were spotted on host bacterial lawn. Vibrio phage Virtus stored at 4 °C for 24 h was used as a control. Stability studies for acidic and alkaline pH conditions were conducted according to the methods described by Pan et al. [22], with some modifications. Briefly, phages were suspended in LB adjusted with 1 M NaOH or HCl (Thermo Fisher Scientific, Branchburg, NJ, USA) to yield a pH range of 1–10, and incubated at 4 °C for 2 h, and then the phage aliquots were serially diluted and spotted on host bacterial lawn to determine the titer and the survival of the phage. The sensitivity of Vibrio phage Virtus to chloroform was determined by exposing ≈10^11^ PFU mL^−1^ of the phage aliquots to 10% chloroform at 4 °C for 1 h, while the stability of the Vibrio phage Virtus against commonly used disinfectants in aquaculture was measured by exposing ≈ 10^11^ PFU mL^−1^ of Vibrio phage Virtus to 0.001% benzalkonium chloride (BKC), 3% hydrogen peroxide (H_2_O_2_), 1% sodium hypochlorite (NaOCl), 70% ethanol (EtOH), and 1% formaldehyde (CH_2_O) at 25 °C for 1 h. Vibrio phage Virtus incubated at 25 °C for 1 h were used as control. Each treatment was serially diluted and spotted on host bacterial lawn. The phage titer was assessed after the agar plates were incubated at 25 °C for 24 h. All assays were conducted in triplicate.

### 2.8. One-Step Growth

One-step growth of Vibrio phage Virtus was determined according to Clokie et al. as described in Misol et al. with some modifications [16,17]. Briefly, 1 mL of host culture inoculated LB broth until it reached exponential phase (≈10^8^ CFU mL^−1^) and was then centrifuged at 13,000 rpm for 3 min. The supernatant was then discarded, and the pellet was washed and resuspended in 1 mL of SM buffer (5.8 g/L NaCl, 2 g/L MgSO_4_, 50 mL 1 M Tris-Cl (pH 7.5) and 2% gelatin, 1 L deionized H_2_O). This step was then repeated twice before the pellet was finally resuspended in 1 mL of LB. The fresh host culture was then inoculated with Vibrio phage Virtus at MOI 0.01. Following incubation for 10 min at 25 °C, the infected *Vibrio harveyi* VH2 culture was transferred to LB with the final volume of 30 mL. At 10 min intervals, 1 mL aliquots were collected from the infected host culture and were centrifuged for 13,000 rpm for 3 min. Subsequently, the supernatants were collected, serially diluted, and spotted on the host bacterial lawn on LB/2 agar plates. The phage titer was assessed after the incubation of agar plates at 25 °C for 24 h. For the assessment of the eclipse period, the same procedure was followed, but instead of centrifuging the samples, chloroform was added. Burst size was calculated as the ratio of the final count of liberated virions at the end of the burst period to the initial count of infected bacterial cells at the beginning of the latent period.

### 2.9. In Vitro Cell Lysis

The in vitro cell lysis of Vibrio phage Virtus against *Vibrio harveyi* VH2 was carried out by loading 180 µL of fresh host bacterial culture in each well of sterile 96-well plates. The plates were then read at OD_600_ using a TECAN microplate reader (Infinite PRO 200) at 25 °C with orbital shaking. A total of 20 µL of Vibrio phage Virtus was then added at MOIs 0.1, 1, 10, and 100 when host culture was at the exponential phase (≈10^7^ CFU mL^−1^). Phages added to LB without host bacteria served as the control. The growth curves of the cultures were then measured every 10 min for 24 h. All assays were performed in triplicate.

### 2.10. DNA Extraction and Purification

The DNA extraction of Vibrio phage Virtus was carried out using the phenol-chloroform method according to Higuera et al. [23]. The extracted DNA was visualized for quality on 1% agarose gel electrophoresis at 80 kV for 1 h with a 50 kbp ladder. Milli-Q^®^ Reference Water (Merck KGaA, Darmstadt, Germany) was used as a negative control. The extracted DNA of Vibrio phage Virtus was then stored in −20 °C.

### 2.11. Genomic Analysis

The genome of Vibrio phage Virtus was sequenced using the DNBSEQ™ sequencer using paired-end technology (PE100) at BGI, Hong Kong. The workflow for library preparation for the platform included fragmentation, size selection, end repair and A-tailing, bubble adaptor ligation, PCR amplification, denaturation, splint circularization, enzymatic digestion and purification, and DNB making. Raw reads were filtered if more than 25% matched the adapter sequence, if more than 50% bases had quality values lower than 20 and if there were more than 3% N in the read. Filtering was completed using the SOAPnuke software. The raw reads were quality inspected and were assembled by Unicycler v0.4.8 in PATRIC [24]. QUAST v4.6.3 [25] and BBMap v38.88 [26] were used to map the reads back to the assembled genome, while PhageTerm was used to predict phage termini [27] through the Galaxy server [28]. RASTk, Glimmer, and GeneMark were used for gene prediction. Sixpack, a naive gene caller, was used as validation to annotate genes that may have been missed by Glimmer. Moreover, potential protein-coding genes were manually checked to ensure the presence of a phage start codon (ATG/GTG or TTG), and a Shine–Dalgarno feature was added to all features that had a detectable match. Proteins of Vibrio phage Virtus were manually annotated using (i) NCBI Basic Local Alignment Search Tool (BLAST) [29] adjusted at non-redundant (nr) protein database, (ii) Gene Ontology [30], (iii) InterPro [31], and (iv) TΜHMM 2.0 [32,33]. Predicted proteins of Vibrio phage Virtus were also manually annotated with NCBI Conserved Domain Database (NCBI CDD) [34]. All ORF predictions and annotations were manually inspected. Integrase, virulence, and antibiotic-resistance-encoding genes in Vibrio phage Virtus were for searched using the INTEGRALL Database webserver [35], Virulence Factor Database (VFDB) [36], and VirulenceFinder and ResFinder webservers [37]. The host *Vibrio harveyi* VH2 genome was analyzed for prophage-like sequences using Phage Search Tool Enhanced Release (PHASTER) [38]. A computational analysis using Bacphlip [39] was conducted in order to assess phage lifestyle on the basis of phage proteome. For protein structural homologies, only probabilities above 90% were accepted for manual protein function assignment of the Vibrio phage Virtus predicted ORFs. All hits were in existing databases with expected E-value below 10^−3^. The genome of Vibrio phage Virtus with annotated predicted ORFs was then visualized in a circular representation with Geneious software (Geneious v9.1, Biomatters, Auckland, Australia) and CGview.

### 2.12. Genome Alignment and Phylogenetic Analysis of Vibrio phage Virtus

The whole proteome of Vibrio phage Virtus was searched for similarity with other phages using the NCBI BLASTP nr protein database. The phage genomes with significant similarities were then downloaded and aligned with Vibrio phage Virtus using the progressiveMauve: Multiple Genome Alignment [40] for analysis of the genomic synteny. Pairwise alignment with of Vibrio phage Virtus with vB_VcaS_HC was conducted using Geneious Alignment with a cost matrix of 65% similarity (5.0/−4.0) on the basis of the Needleman and Wunsch (1970) and Smith and Waterman (1981) algorithms [41,42]. ViPTree was used to investigate the taxonomic position of Virtus and its host [43]. MEGA X was used to analyze the phylogeny and molecular evolution of the novel phage in comparison with other *Vibrio* phages [44]. Eighteen large terminase subunits of described Vibrio phages were downloaded from the NCBI database and were aligned with the large terminase subunit of Vibrio phage Virtus using MUSCLE algorithm [45]. Gaps in the amino acid sequence alignments were trimmed. A maximum likelihood phylogenetic tree was constructed using the TN93 model [46] with bootstrap test = 1000. The tree was visualized using the Interactive Tree of Life web server [47].

### 2.13. In Vivo Phage Therapy Trial in Gilthead Seabream Larvae

Gilthead seabream (*Sparus aurata*) larvae were selected as a model to assess the therapeutic potential of Vibrio phage Virtus. Gilthead seabream eggs at the same developmental stage were obtained from HCMR hatchery, washed three times with sterile sea water, and placed individually in a 96-well microplate (1 egg/well) containing 200 μL sterile sea water. After one day of incubation, the quality of eggs was evaluated according to Panini et al. [48]. The challenge test started when eggs were hatched.

Bacteria used in the challenge test were grown in LB overnight and diluted 1:100 in fresh LB. After a 2 h incubation at 25 °C, cells were centrifuged and washed twice with buffer A (saline 0.9%, MgCl_2_ 10 mM). The bacterial suspensions were adjusted to ≈10^7^ CFU mL^−1^ with buffer A. No treatment occurred in the first group of larvae. The second group was treated with Vibrio phage Virtus alone (without addition of bacteria) at an approximate concentration of 10^8^ PFU ml^−1^ and served as a negative phage control. The third group was treated with 10^6^ CFU ml^−1^ of a *Phaeobacter piscinae* S26 strain, which has probiotic properties and served as a control to assess the effect of the addition of the same quantity of non-pathogenic bacteria on the viability of the larvae. The fourth group was treated with 10^6^ CFU mL^−1^ *Vibrio harveyi* VH2. The fifth and sixth groups were treated with 10^6^ CFU mL^−1^ *Vibrio harveyi* VH2 and Vibrio phage Virtus at 10 ΜOΙ. A second dose of Vibrio phage Virtus was administered the following day in the sixth group, at the same MOI. Phage suspensions were treated with 10% (*w/v*) PEG overnight at 4 °C to remove possible endotoxins in the phage lysate and were diluted in SM buffer (NaCl 100 mM, MgSO_4_7H_2_O 8 mM, Tris-Cl 1 M; pH 7.5). The phage titer was also determined prior to the experiment with double agar assay. Phage suspensions were added to the corresponding treatments two hours after infection. In addition, all controls were treated the same way, but instead of phage lysate, saline 0.9% was added to each well. The survival of gilthead seabream larvae was monitored daily for the following five days. A Kaplan–Meier survival curve was then constructed using GraphPad Prism version 9.0.0 for Windows (GraphPad Software, San Diego, CA, USA).

### 2.14. Statistical Analysis

One-way ANOVA was performed for the thermal and pH stability, and effects of organic solvents assays along with Dunnett’s multiple comparison test [49]. Tukey’s HSD post hoc test [50] was used as a multiple comparison tool after ANOVA was performed. Kaplan–Meier survival analysis [51] was performed for the in vivo phage therapy trial in gilthead seabream larvae. All statistical analyses were carried out using GraphPad Prism version 9.0.0 for Windows, GraphPad Software, San Diego, CA, USA).

### 2.15. Data Availability

The genome sequence of phage Vibrio phage Virtus is available in GenBank under accession number OK381870. The associated BioProject and BioSample accession numbers are PRJNA764828 and SAMN21529761, respectively.

## 3. Results

### 3.1. Isolation and Morphology of Vibrio phage Virtus

**Figure 1:**
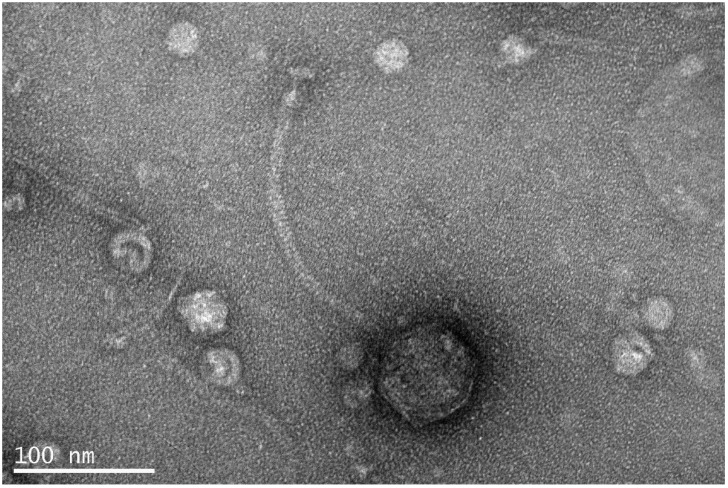
Transmission electron microscopy picture of Vibrio phage Virtus showing a typical morphology of siphoviruses.

Vibrio phage Virtus was isolated from fish tank water collected from the broodstock section of the aquaculture facilities of the Institute of Marine Biology, Biotechnology and Aquaculture of the Hellenic Centre for Marine Research in Heraklion, Greece, against *Vibrio harveyi* VH2 [15]. Throughout the propagation steps, Vibrio phage Virtus showed a consistent plaque morphology producing pinhole-type plaques with a diameter of 0.42 ± 0.05 mm (*n* = 40). Transmission electron microscopy (TEM) showed that Vibrio phage Virtus has a long non-contractile, conspicuously striated tail and an icosahedral capsid (Figure 1), morphologically consistent with the *Siphoviridae* family. The phage capsid was 70 ± 05 nm in width, and the tail was 220 ± 10 nm long and 12 ± 2 nm wide. Finally, the baseplate had a width of 20 ± 02 nm and a length of 13 ± 01 nm.

### 3.2. Host Range and Efficiency of Plating (EOP) of Vibrio phage Virtus against Multiple Antibiotic Resistant Strains

Vibrio phage Virtus was able to infect 13 out of 25 strains tested (Table 2). It infected 8 of the 16 strains of *V. harveyi*; the single strains of *V. parahaemolyticus*, *V. campbellii*, and *V. mediterranei*; and one out of two strains of *V. owensii*. The strains of *V. alginolyticus*, *V. rotiferianus*, and *V. splendidus* tested were not susceptible to the phage. EOP of Vibrio phage Virtus was high for four strains of *V. harveyi* (SA 1.2, VhP1 Spl, VH2, Kef 75), and moderate for five strains of other *Vibrio* spp. (VhSerFrE, Vh28, L. SUSI, SA 6.2, VIB391). All strains used in the assay are from the HCMR collection and have been identified to species level through sequencing.

### 3.3. Thermal and pH Stability of Vibrio phage Virtus and Exposure to Organic Solvents and Common Disinfectants

**Figure 2:**
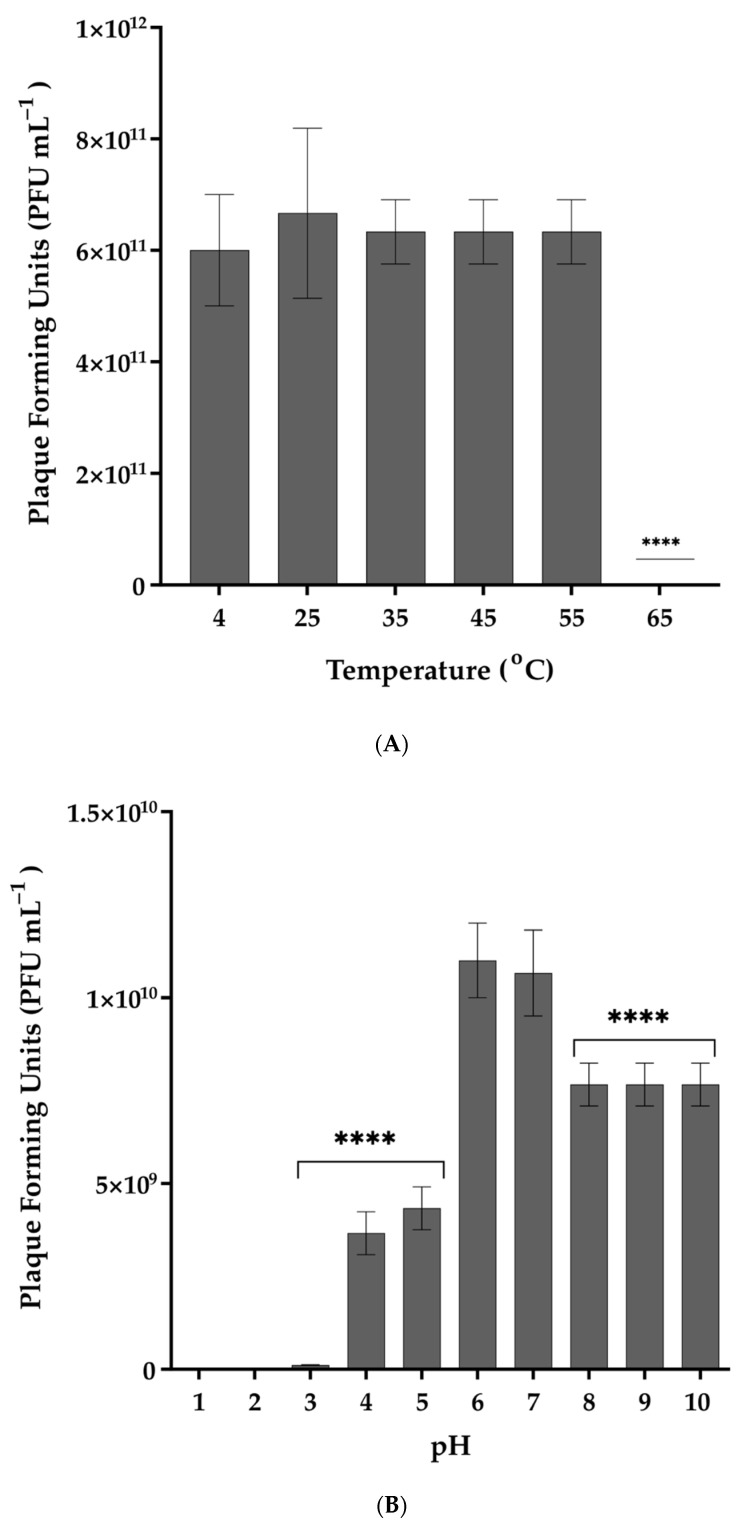
(**A**) Effect of different temperatures on the stability of Vibrio phage Virtus. Incubation at 4 °C was used as control. (**B**) Effect of pH in the stability of Vibrio phage Virtus. Incubation with pH = 7 was used as control. Phage titer was measured against *V. harveyi* VH2. Error bars were shown for the mean of *n* = 3. Statistical significance indicated by **** at *p* < 0.0001.

**Figure 3:**
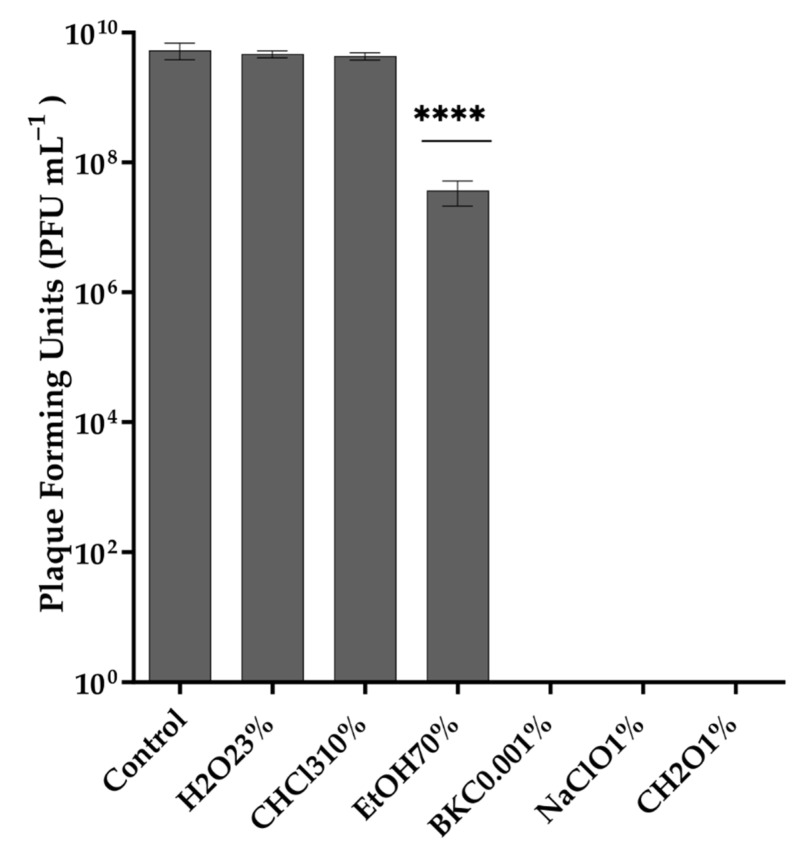
Effect of different organic solvents to the stability of Vibrio phage Virtus. Incubation with LB was used as control. Phage titer was measured against *V. harveyi* VH2. Error bars were shown for the mean of *n* = 3. Statistical significance indicated by **** at *p* < 0.0001 compared to the control.

Exposure to different temperatures showed that Vibrio phage Virtus was stable between 4 and 55 °C (Figure 2a). No statistically significant difference (F(4, 10) = 0.1923, *p* = 0.9369) of its titer was observed at the temperatures assessed, while a complete inactivation was observed from 65 °C and above. The optimum pH of Vibrio phage Virtus was 6 (Figure 2b). Complete inactivation was observed at low pH values, while statistically significant reduction of the titer was observed at pH 3, 4, 5, 8, 9, and 10 compared to the control (F(9, 20) = 150.5, *p* < 0.001). A one-way ANOVA was performed to compare the effect of 6 different organic solvent solutions and common disinfectants to phage titer (F(6, 14) = 46.08, *p* < 0.001) (Figure 3). Hydrogen peroxide and chloroform did not affect Vibrio phage Virtus titer (*p* = 0.6609, *p* = 0.2975). However, there was a significant reduction when the phage was exposed to 70% ethanol (*p* < 0.001). Complete inactivation was observed in BKC, NaClO, and CH_2_O.

### 3.4. One-Step Growth of Vibrio phage Virtus

**Figure 4:**
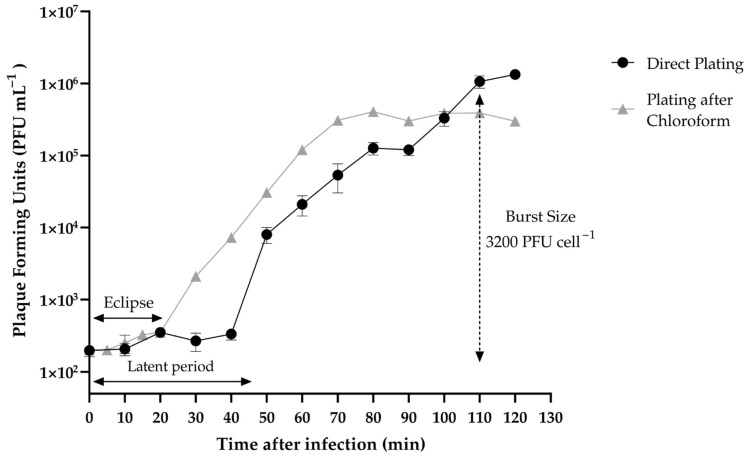
One-step growth of Vibrio phage Virtus measured against *V. harveyi* VH2 at multiplicity of infection (MOI) 0.01. Error bars were shown for the mean of *n* = 3.

One-step growth assay (Figure 4) showed that Vibrio phage Virtus has a latent phase of 40 min and an eclipse phase of 20 min. The rise phase was estimated between 40 and 110 min. The plateau phage was reached at 110 min. In this assay, the burst size of Vibrio phage Virtus was 3200 PFU per cell.

### 3.5. In Vitro Cell Lysis

**Figure 5:**
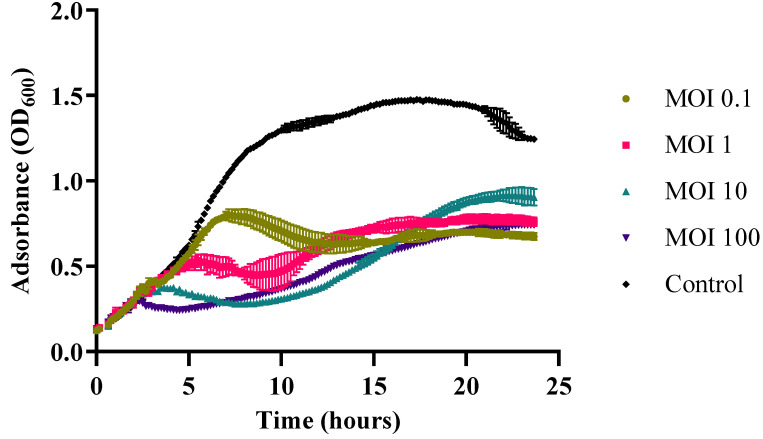
In vitro lysis of Vibrio phage Virtus against *V. harveyi* VH2 at MOIs 0.1, 1, 10, and 100 for 24 h. Bacterial growth indicated by the absorbance (OD_600_) read. Error bars were shown for the mean of *n* = 3.

In vitro lysis assay with *Vibrio harveyi* VH2 showed that Vibrio phage Virtus was able to lyse the host bacterial population from MOI 0.1 to 100 after 24 h of incubation (Figure 5). The growth of the bacteria treated with the Vibrio phage Virtus was inhibited at 7, 5, 3.5, and 2 h post infection for MOIs 0.1, 1, 10, and 100, respectively, and a significant reduction of their titer compared to the untreated control was maintained until the end of the experiment. The titer of *V. harveyi* VH2 was reduced by 40–50% at MOIs 0.1, 1, and 100 compared to the control group over a 24 h period.

### 3.6. Whole Genome Sequencing and Assembly

Genome sequencing of Vibrio phage Virtus resulted in 6,207,226 clean reads with an average read length of 100 bp and 100% correct base calls. The GC content was 47.42%. Genome assembly resulted in a single contig with a minimum genome coverage of 5×. Genome length was 82,960 bp with coverage depth of 7912.21×. According to PhageTerm analysis, the Vibrio phage Virtus genome did not have any termini and was found to be terminally redundant and circularly permuted.

### 3.7. Genomic Features of Vibrio phage Virtus

The genome size of Vibrio phage Virtus is 82,960 bp. The genome arrangement was dense, as suggested by the 1.53 genes per kbp. A total of 127 ORFs were identified with Rapid Annotation using Subsystem Technology (RASTk) server, Glimmer.hmm 2.0, and GeneMark. Comparison of the predicted ORFs showed that all ORFs called by Glimmer.hmm 2.0 and GeneMark were also called by RASTk. Manual inspection of each predicted ORF and gap between ORFs, as well as subsequent alignment in the NCBI nr database, validated that the 127 predicted ORFs were present in the Vibrio phage Virtus genome. No tRNA was found in the genome. A total of 119 ORFs used a start codon of ATG, 6 ORFs used GTG, and 2 used TTG. A search of the NCBI nr database showed that 121 ORFs (95.27%) had significant hits (expected value ≤10^−3^) with an average similarity of 85.62%. A total of 109 ORFs (85.8%) were determined to have best hits with Vibrio phage vB_VcaS_HC MK559459.1, which infects *V. campbellii*, while 12 ORFs (9.44%) had the best hits with another six similar Vibrio phages: Vibrio phage 1 (JF713456.1), Vibrio phage Ares1 (MG720309.1), Vibrio phage Thalassa YP (MG649967), Vibrio phage vB_ValS_PJ32 (MT735629.1), Vibrio phage vB_VhaS-a (KX198615.1), and Vibrio phage vB_VpaS_VP-RY-9 (MW411580.1). In addition, protein structural homolog search for the predicted ORFs showed 9 hits in the Gene Ontology database, 7 hits with InterPro, and 24 hits with the NCBI CDD. Overall, 43 (33.8%) ORFs were annotated on the basis of amino acid sequence and protein structural homologies. No homologs of integrase, virulence, or antibiotic-resistance-encoding genes were found in Vibrio phage Virtus. Computational analysis based on phage proteome revealed that there is 92.5% probability that Vibrio phage Virtus follows a lytic lifestyle.

### 3.8. Genomic Arrangement and Functional Annotations of Vibrio phage Virtus

**Figure 6:**
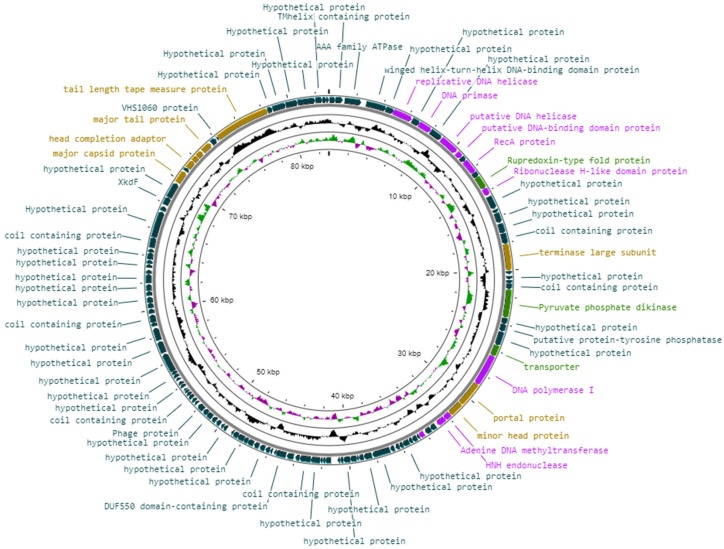
Visual representation of the Vibrio phage Virtus genome in which the genome GC content is shown by the inner black line and the GC skew by the inner purple/green line. The predicted ORFs are shown as arrows. The color of the ORFs refers to annotated biochemical function: phage assembly proteins (brown); DNA-replication-, repair-, and recombination-associated proteins (purple); auxiliary metabolic proteins (light green); hypothetical (dark green).

Generally, the genome of Vibrio phage Virtus did not have any modular arrangement (Figure 6). However, some genes encoding for head and tail proteins (ORF 112, ORF 114, ORF 115, ORF 117, ORF 120) were arranged in subclusters as well as some genes encoding for DNA replication and nucleotide metabolism proteins (ORF 6, ORF 8, ORF 10, ORF 11, ORF 13, ORF 16). Genes that were functionally annotated are shown in Table 2.

#### 3.8.1. Phage Structural Proteins

Proteins required for phage assembly, including major tail protein (ORF 117), major capsid protein (ORF 112), tail length tape measure protein (ORF 120), tail-completion protein (ORF 116), head completion adaptor (ORF 114), neck protein (ORF 115), portal protein (ORF 33), and minor head protein (ORF34). In addition, the large terminase subunit involved for DNA packaging for tailed phages was identified at ORF 22.

#### 3.8.2. DNA Replication, Repair, and Recombination

Proteins for DNA replication, recombination, and repair were also identified: RecA (ORF 13), HNH endonuclease (ORF 30), DNA polymerases (ORF 32, 39), DNA helicases (ORF 6, 10, 89), DNA primase (ORF 8), and other regulatory elements (ORF 11, 16, 36).

#### 3.8.3. Miscellaneous Proteins

Several transmembrane proteins were detected (ORF 2, 37), including a possible K+-dependent Na^+^/Ca^+^ exchanger at ORF 111 (Table 3). Additionally, auxiliary metabolic genes were detected; rubredoxin-type fold protein (ORF 15), a transporter (ORF 31), and a gene coding the pyruvate phosphate dikinase (PPDK), whose product plays a key role in the Embden–Meyerhof–Parnas (EMP) glycolytic pathway (ORF 27).

### 3.9. Genomic Synteny of Vibrio phage Virtus with Other Similar Phages

**Figure 7:**
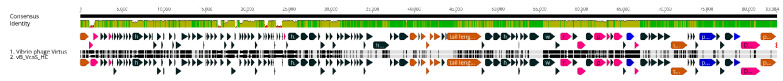
Pairwise alignment of Vibrio phage Virtus with vB_VcaS_HC. From the top, the first bar represents mean pairwise identity over all nucleotide pairs (green: 100% identity, brown: at least 30% and under 100% identity, red: below 30% identity). Predicted ORFs are shown by arrows. The color of the ORFs refers to annotated biochemical function; phage assembly proteins (orange), DNA-replication-, repair-, and recombination-associated proteins (purple); auxiliary metabolic proteins (blue); hypothetical (dark green).

**Figure 8:**
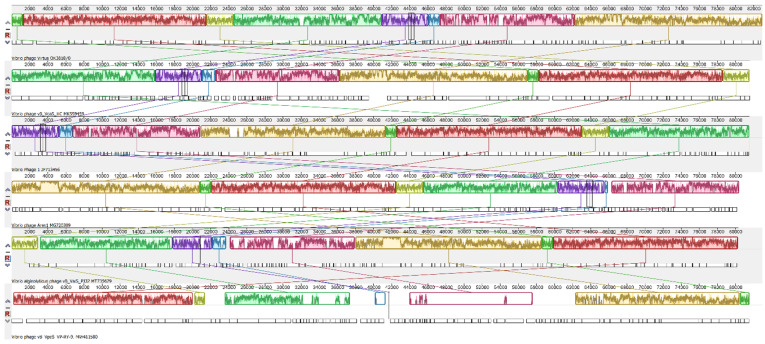
Whole genome alignment with progressive MAUVE of Vibrio phage Virtus with similar phages. From the top is Vibrio phage Virtus, vB_VcaS_HC, Vibrio phage 1, Vibrio phage Ares1, vB_ValS_PJ32, and vB_VpaS_VP-RY-9. The colored collinear blocks indicate homologous regions between genome sequences, while the height of the similarity profile in the collinear blocks indicate average level of conservation in the regions of the genome sequence. Inverted blocks indicate homologous regions that align in the complement orientation.

Pairwise alignment between Vibrio phage Virtus and vB_VcaS_HC showed that they have a genetic identity of 94.2% (Figure 7). The areas coding the proteins required for the phage structural assembly were generally conserved; however, there were significant nucleotide disagreements in genes who relate to DNA replication and nucleotide metabolism, i.e., ORF 10, ORF 11, ORF 15, and in areas coding miscellaneous proteins. A gene coding a homing endonuclease (ORF 35), two genes coding hypothetical proteins (ORF 86, ORF 87), and a non-coding area were present in the Vibrio Virtus genome but not in vB_VcaS_HC. The Vibrio phage Virtus had the highest degree of genomic synteny with vB_VcaS_HC (Figure 8) sharing eight collinear blocks. The longest shared collinear block had a sequence length almost 20,000 bp. Furthermore, the common collinear blocks had similar genomic arrangements and shared high DNA sequence similarities. Alignment with another three similar vibrio phages: Vibrio phage 1, Vibrio phage Ares1, and *Vibrio alginolyticus* phage vB_ValS_PJ32, also showed eight shared collinear blocks of similar length with high genomic synteny and sequence similarities. On the contrary, both Vibrio phage Virtus and vB_VpaS_VP-RY-9 shared six collinear blocks, but with very low sequence similarities.

### 3.10. Phylogenetic Analysis

**Figure 9:**
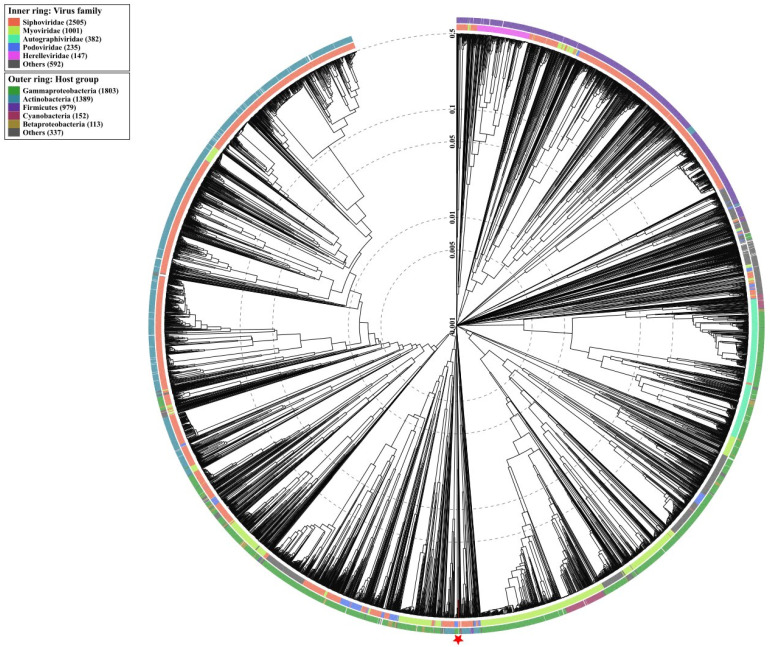
Determination of taxa and host group for Vibrio phage Virtus according to the proteomic tree produced by VIPTree. Vibrio phage Virtus was determined to belong to the Siphoviridae family and to infect Gammaproteobacteria group (red star and line). Vibrio phage Virtus (asterisk) proteome was compared with 4892 dsDNA phage proteomes. The branch length scale was calculated as log values. The inner and outer ring indicate the taxonomic virus family and host group, respectively.

Wide genome proteomic tree analysis confirmed that Vibrio phage Virtus belongs to the *Siphoviridae* taxonomic family (Figure 9). In addition, Vibrio phage Virtus was predicted to infect hosts from the Gammaproteobacteria class, which includes the Vibrionaceae family.

**Figure 10:**
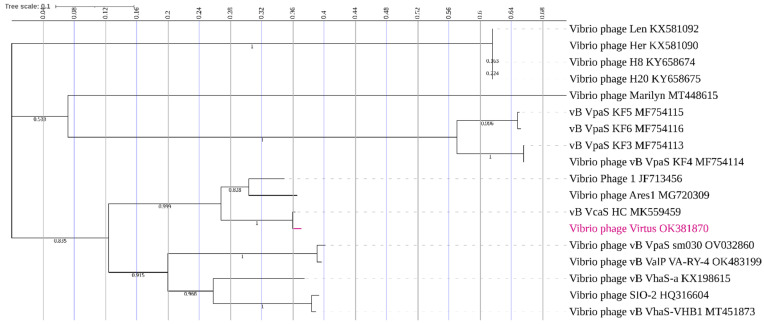
Phylogenetic tree of Vibrio phage Virtus with other Vibrio phages. The large terminase subunits of similar phages were downloaded from the NCBI database and aligned using MUSCLE, and a maximum likelihood (bootstrap = 1000) phylogenetic tree was constructed using MEGA X. The tree was visualized using the Interactive Tree of Life (ITOL). The bootstrap support value is denoted in each branch.

Phylogeny using large terminase subunits of vibrio phages (Figure 10) showed that Vibrio phage Virtus has a recent common ancestor with vB_VcaS_HC. Moreover, Vibrio phage Virtus has a high bootstrap support (100%) with Vibrio phage 1 and Vibrio phage Ares1, indicating that they share a common evolutionary history. In addition, the branch length is proportional to the amount of evolutionary divergence, and hence Vibrio phage Virtus and vB_VcaS_HC phages share a similar number of amino acid substitutions in their large terminase subunit since diverging from their common ancestor.

### 3.11. In Vivo Phage Therapy in Gilthead Seabream Larvae

**Figure 11:**
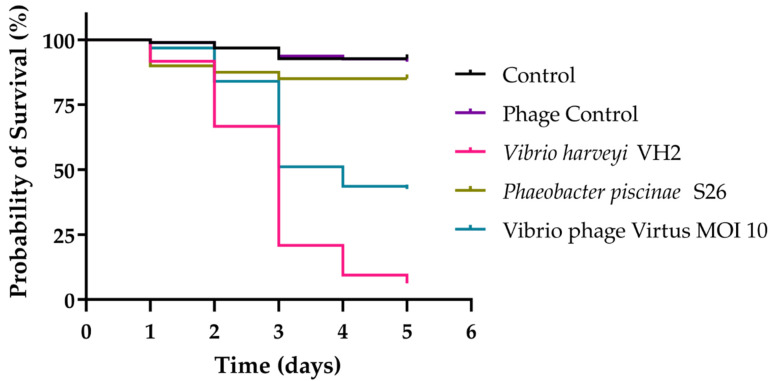
Survival of gilthead seabream larvae infected with *V. harveyi* VH2 in an experimental phage therapy trial during a period of 5 days. Gilthead seabream larvae that were infected with VH2 were inoculated with Vibrio phage Virtus with different multiplicities of infection (MOI) at 2 h post-infection. *Phaeobacter piscinae* S26 were used to evaluate the effect of non-pathogenic bacteria at the same concentration to fish larvae.

In vivo phage therapy trials with gilthead seabream larvae were conducted to assess the efficacy of Vibrio phage Virtus in controlling *Vibrio harveyi* VH2 (Figure 11). VH2 was found to be very pathogenic, significantly reducing the survival of larvae to just 6% compared to the control group in which 92% of larvae survived during the 5-day trial (*X*^2^ (1, 192) = 148.6, *p* < 0.001). Survival of gilthead seabream larvae was significantly increased when treated with Vibrio phage Virtus at a MOI of 10 compared to the group treated with *V. harveyi* VH2 (*X*^2^ (1, 190) = 33.4, *p* < 0.001). Moreover, no significant reduction was observed between the single dose and the two doses of treatment (data not shown). The phage control group (no bacteria added) also had no significant difference compared to the control (*X*^2^ (1, 191) = 0.07865, *p* = 0.7791), indicating the safety of the phage suspension and possibly the absence of endotoxins (Figure 10).

## 4. Discussion

*Vibrio harveyi* outbreaks are increasing, as climate change becomes more imminent, threatening a broad range of marine organisms such as abalones, shrimps, corals, and various fishes [52,53,54,55] and leading to severe economic and production losses in aquaculture worldwide [56,57]. The biggest problem associated with these outbreaks is that many strains are highly resistant to antibiotic treatments [58,59,60]. Because of this, an increasing number of studies aiming to control vibriosis have been conducted by employing phages as therapeutic agents. To our knowledge, 21 bacteriophages have been previously isolated against *V. harveyi*, including 16 siphoviruses [61,62,63,64,65,66], 4 myoviruses [17,62,67,68], and 1 podovirus [69]. Two bacteriophages, VHML and Siphophage 1 VHS1, were found to be temperate [66,68], while the rest are considered to be lytic. Here, we isolated a novel lytic bacteriophage, Vibrio phage Virtus, against *V. harveyi* VH2, and tested its efficacy as a potential candidate for therapy.

Whole sequence homolog search and pairwise alignment revealed that a Vibrio phage, vB_VcaS_HC, which infects *Vibrio campbelii*, shared a high similarity with Vibrio phage Virtus. Both methods yielded 94.2% genetic identity between the phages. The threshold to distinguish two different species is 95%, and thus Vibrio phage Virtus probably belongs to a novel species of the *Siphoviridae* family [70]. Both phages shared similar genomic arrangements with nucleotide similarities according to genomic synteny analysis. Interestingly, Vibrio phage Virtus was isolated in Heraklion, Greece, while vB_VcaS_HC was isolated in Qingdao, China. This suggests that this particular phage has a rather wide geographical distribution. Phage geographical distribution depends on the abundance and metabolic state of the host, since phage survival depends on the presence of susceptible hosts [71]. Thus, a phage with wide geographical distribution indicates that either its host is ubiquitous or that the phage has a broad host range. Moreover, specific phage traits such as latent period and burst size may also influence phage dispersal and what geographical patterns it follows [72]. High burst size and long latent period improve the probability of a successful dispersal and are indicatives of a cosmopolitan phage. In our case, Vibrio phage Virtus was capable of infecting hosts from different species, unlike most phages, which are usually species-specific [73]. On the contrary, vB_VcaS_HC had a very narrow host range. However, no direct comparison can be made, since the host range is related to the bacterial strains used, which were different in the two studies. It is suggested that a broad host range is an important evolutionary trait for phages [74], although often with a decreased virulence as a cost, which reflects the antagonistic pleiotropy [75]. However, phages with a broad spectrum of hosts are desirable for therapy, especially for pathogens that are abundant and diverse such as the vibrios [76,77].

Vibrio phage Virtus was found to have an unusually large burst size. To date, only a few phages have been reported to have such large burst sizes in all of dsDNA phages [22,78,79]. The eclipse period was estimated to be longer than the average of most phages, which is 5–15 min [80], and in combination with the long latent period, could possibly lead to high virion productions due to multiple reproduction cycles [81]. However, other factors may also affect the burst size, including the host metabolic activity, ambient environment, and the protein synthesis machinery of the host bacteria [22,82,83], and hence the molecular mechanism associated with the large burst size needs further investigation.

Horizontal gene transfer (HGT) occurs regularly between phages and bacteria populations either by generalized or specialized transductions [69,84,85]. Vibrio phages have occasionally been associated with inducing virulence in their hosts [68,86], and hence a comprehensive profiling of their genomic traits is required before proceeding to therapeutic application. Only 4 of the 21 phages against *V. harveyi* that were isolated in previous studies have been sequenced and characterized genomically [17,63,86,87]. The genome sizes of these four phages vary between 48 and 286 kbp, including a jumbo bacteriophage Vb_VhaM_pir03. In the Vibrio phage Virtus genome, no integrase, virulence, or antibiotic-resistance-encoding genes were detected. Moreover, no prophage induction occurred when host mutants with a phage-resistant phenotype were exposed to UV radiation, further supporting the lytic lifestyle of the novel phage. The Vibrio phage Virtus genome is absent of any termini, is circularly permuted, and is terminally redundant, which suggests a headful packaging mechanism [88,89]. An auxiliary metabolic gene coding the pyruvate phosphate dikinase (PPDK) whose product plays a key role in the Embden–Meyerhof–Parnas (EMP) glycolytic pathway was present [90]. PPDK is not commonly found in phages, yet it has been reported before in some vibrio siphoviruses [10,62,84]. Phages that contain auxiliary metabolic genes have mechanisms to manipulate host metabolism into their own benefit [91]. For example, the lytic bacteriophage KVP40 genome includes ORFs that encode proteins that facilitate precursor transport and synthesis of NAD^+^ in the pyridine nucleotide salvage pathway [92]. Moreover, studies have shown that marine viruses genomes, isolated in nutrient-limited environments, were rich in auxiliary metabolic genes compared to the ones isolated in nutrient-rich environments [93], indicating a strong association between phage auxiliary metabolic genes and host resource uptake. Genes encoding phosphorus uptake regulation such as PhoH have been found in Vibrio phages [94], and it has been suggested that they are being used in order to force the host to increase phosphorus acquisition in order to be used during phage DNA replication. Taking this into consideration, it is likely that the phage PPDK gene is co-expressed during infection, increasing host energy uptake, which is ultimately directed to the production of more virions. The presence of auxiliary metabolic genes in the Vibrio phage Virtus genome can also be linked to a possible widespread distribution, since they can lead to a higher burst size and can thereby expand dispersal [72,95].

As shown in stability assays, Vibrio phage Virtus can withstand a wide range of temperatures and pH values, which is very practical for phage therapy. In addition, we showed that Vibrio phage Virtus can be completely inactivated with various organic solvents if this is required to reduce the risk of unwanted dissemination to the environment. In vitro assay showed that Vibrio phage Virtus was able to efficiently reduce the host bacterial populations at different MOIs. The fact that it was able to lyse the bacteria in low MOIs offers a practical advantage for the application in the aquaculture settings, since the required phage quantity is relatively low. However, after 15 h, the host bacterial population started to rise again, suggesting the emergence of resistance, possibly due to the intense selective pressure [96]. Phage resistance is a concerning issue in phage therapy [97] since bacteria populations have various protection mechanisms against phages [98]. The combination of different phages, phage cocktails, has been suggested as a workaround, a practice that has yielded promising results [99,100,101].

Several studies have shown the successful in vivo application of phages to treat vibriosis in various animal models [17,61,102]]. Wang et al. has shown that vB_VhaS-tm managed to improve survival of abalone by 70% in seven days, while Misol et al. showed that vB_VhaM_pir03 improved *Artemia* nauplii survival by 15–20% in 48 h [17,102]. Moreover, the survival of giant tiger prawn (*Penaeus monodon*) was immensely higher when treated with phages compared to antibiotic treatment and the control, as shown by Vinod et al. [61]. Here, in vivo phage therapy trials in gilthead seabream larvae showed that a single dose of Vibrio phage Virtus significantly improved the survival of the larvae by 35% compared to the untreated population. As Levin and Bull [103] suggested, phages decrease bacterial load enough to be eliminated by the fish immune system. In this case, we suggest, that Vibrio phage Virtus decreases the bacterial population in levels that are no longer pathogenic by reducing the colonization of *Vibrio harveyi* in the larvae skin [104]. Moreover, we showed that a second dose of Vibrio phage Virtus made no difference in the survival of the larvae. It is possible that the phage and the bacteria population had already reached an equilibrium, known as the carrier state. In this state, bacterial populations are heterogenous, as they contain subpopulations in which phages are stably maintained within the host rather than committing to lysis or subpopulations that are resistant to phages and maintain the sensitive population [105]. The molecular mechanism behind the resistance of carrier state bacterial subpopulations is very intriguing and worthy of further investigation. Preliminary experiments for the characterization of resistant mutants (not included here) of host developed in this study revealed a fitness cost to the bacteria (data not shown), which suggests that the defense mechanism is more likely related to cell surface modifications [106,107]. However, this is a mere speculation, since other defense mechanisms have been reported before, such as the acquisition of spacers matching phage genomic material [105]. The emergence of resistance could limit the therapeutic potential of Vibrio phage Virtus, and therefore its synergistic effect with other *Vibrio* phages is being pursued. However, no fully characterized phage infecting *V. harveyi* VH2 was available at the time of the study, and thus a comparative or a synergistic treatment with another phage was not possible. Carrier state often results in less pathogenic bacteria populations, as shown in previous studies [105,108]. Furthermore, no significant mortalities were detected in the group treated with only the phage suspensions, indicating the safety of Vibrio phage Virtus to the fish larvae. Although gilthead seabream larvae have been used as an in vivo model to study the therapeutic efficacy of the Vibrio phage Virtus, it should be noted that given the importance of this fish species for the Mediterranean aquaculture [109] and the high prevalence of vibriosis caused by *V. harveyi* [7], the practical usability of Virtus but also similar phages in commercial aquaculture is evident. Of course, there are several issues that remain to be resolved before phage therapy for aquaculture becomes a common practice including regulatory, mass production of phages and resistance development by the bacteria.

In conclusion, we present a comprehensive genomic and biological characterization of Vibrio phage Virtus as a potential and suitable candidate for the biocontrol of *Vibrio harveyi* infections. High virion production and broad host range are the main biological characteristics of Vibrio phage Virtus. As for its genomic profile, Vibrio phage Virtus lacks genes associated with virulence, antibiotic resistance, and transduction potential. On the contrary, its genome contains genes with multiple diverse functions, i.e., PPDK gene, that may contribute to the efficacy of Vibrio phage Virtus. An in vitro assay showed that Vibrio phage Virtus was able to control the host population even at very low MOIs, which favors its practical use in applied therapy. Ultimately, the survival of gilthead seabream larvae challenged with *V. harveyi* was significantly increased when treated with Vibrio phage Virtus, further supporting its effectiveness.

## Figures and Tables

**Table 1:** Bacterial strains used in the study.

Strain ID	Species	Country	Location	Date	Host
DSM 19623	*V. harveyi*	USA	Massachusetts	-	*Talochestria capensis*
SA 2.1	*V. harveyi*	Saudi Arabia	Red Sea	July 2019	*Sparus aurata*
DSM 2171	*V. alginolyticus*	Japan	-	-	*Trachurus trachurus*
Gal 90	*V. harveyi*	Greece	Central Greece	September 2020	*Sparus aurata*
Vh No22	*V. harveyi*	Greece	Ionian Islands	September 2015	*Dichentrachus labrax*
Kef 62	*V. harveyi*	Greece	Ionian Islands	July 2020	*Dichentrachus labrax*
Kef 75	*V. harveyi*	Greece	Ionian Islands	July 2020	*Dichentrachus labrax*
Gal 56	*V. harveyi*	Greece	Central Greece	May 2020	*Dichentrachus labrax*
Gal 77	*V. harveyi*	Greece	Central Greece	July 2020	*Sparus aurata*
Gal 72	*V. harveyi*	Greece	Central Greece	June 2020	*Dichentrachus labrax*
Gal 94	*V. harveyi*	Greece	Central Greece	September 2020	*Sparus aurata*
L. SUSI	*V. parahaemolyticus*	Philippines	Philippines	April 2018	Shrimp
V1	*V. alginolyticus*	Greece	-	May 2018	*Sparus aurata*
LAR194	*V. mediterranei*	Greece	Central Greece	May 2020	*Artemia* nauplii
SM1	*V. harveyi*	Greece	Central Greece	-	*Seriola dumerili*
MAN113	*V. splendidus*	Greece	Saronikos Gulf	September 2019	*Seriola dumerili*
Varv A4/1.1	*V. harveyi*	Greece	Central Greece	July 2019	*Sparus aurata*
VH2	*V. harveyi*	Greece	Crete	2007	*Seriola dumerili*
VhP1 Liv	*V. harveyi*	Greece	Crete	April 2015	*Seriola dumerili*
VhP1 Spl	*V. harveyi*	Greece	Eastern Aegean	September 2015	*Dichentrachus labrax*
DY05	*V. owensii*	Greece	Eastern Aegean	September 2015	*Dichentrachus labrax*
SA 6.2	*V. owensii*	Saudi Arabia	-	July 2019	*Oreochromis niloticus*
VIB391	*V. campbellii*	Thailand	-	October 2016	Shrimp
Kef 56	*V. rotiferianus*	Greece	Ionian Islands	May 2020	*Dichentrachus labrax*
VhSerFre	*V. harveyi*	Greece	Crete	August 2015	*Seriola dumerili*
Phaeobacter S26	*Phaeobacter piscinae*	Greece	Euboea	July 2013	*Artemia* nauplii

**Table 2:** Host range and efficiency of plating of Vibrio phage Virtus against selected Vibrio spp.

Efficiency of Plating of Vibrio Phage Virtus									
Species/Strain	Host Range								EOP
	10^0^	10^−1^	10^−2^	10^−3^	10^−4^	10^−5^	10^−6^	10^−7^	(%)
	*Vibrio harveyi*								
SA 2.1	++	++	++	+	+	+	+	-	High
Varv A4/1.1	-	-	-	-	-	-	-	-	NF
DSM 19623	++	+	+	+	+	-	-	-	Medium
Vh No22	-	-	-	-	-	-	-	-	NF
VhP1 Spl	+++	++	++	++	++	++	+	+	High
VH2	++++	+++	+++	+++	+++	+++	+++	+++	High
VhSerFre	++++	+++	+++	-	-	-	-	-	Medium
VhP1 Liv	-	-	-	-	-	-	-	-	NF
Kef 75	+++	+++	++	++	++	++	+	+	High
SM1	+	-	-	-	-	-	-	-	Low
Gal 56	+	-	-	-	-	-	-	-	Low
Gal 77	-	-	-	-	-	-	-	-	NF
Gal 94	-	-	-	-	-	-	-	-	NF
Gal 72	-	-	-	-	-	-	-	-	NF
Kef 62	-	-	-	-	-	-	-	-	NF
Gal 90	-	-	-	-	-	-	-	-	NF
	*Vibrio parahaemolyticus*								
L.SUSI	++++	+++	-	-	-	-	-	-	Medium
	*Vibrio alginolyticus*								
DSM2171	-	-	-	-	-	-	-	-	NF
V1	-	-	-	-	-	-	-	-	NF
	*Vibrio mediterranei*								
LAR194	+++	+	+	-	-	-	-	-	Low
	*Vibrio splendidus*								
MAN113	-	-	-	-	-	-	-	-	NF
	*Vibrio owensii*								
SA 6.2	++++	+++	+++	++	+	-	-	-	Medium
DY05	-	-	-	-	-	-	-	-	NF
	*Vibrio campbellii*								
VIB391	++++	+++	+++	++	+	-	-	-	Medium
	*Vibrio rotiferianus*								
Kef56	+	-	-	-	-	-	-	-	Low

EOP: efficiency of plating; NF: no plaque formation; ++++: single large clearing zone +++: substantial turbidity throughout clearing zone; ++: ≥20 small plaques; +: <20 small plaques; high: EOP > 10.0%; medium: 0.5% < EOP < 9.9%; low: EOP < 0.5%.

**Table 3:** Summary table of Vibrio phage Virtus ORFs that were annotated with relevant information on the basis of significant amino acid sequences and protein structural homologies (E-value ≤ 10^−3^).

	Predicted Functions	Start	End	Length	Direction	InterPro	NCBI CDD Best Hit	E-Value
ORF1	Hypothetical protein	44	478	434	Forward		pfam18925|DUF5675	7.64 × 10^−30^
ORF2	TMhelix containing protein	480	1019	539	Forward			
ORF3	AAA family ATPase	1143	2366	1223	Forward		cl38936|P-loop_NTPase super family	2.77 × 10^−38^
ORF4	Winged helix-turn-helix DNA-binding domain protein	2753	4246	1493	Forward		cl41463| PspC_subgroup_2 super family	6.13 × 10^−3^
ORF5	Hypothetical protein	4246	4839	593	Forward			
ORF6	Replicative DNA helicase	4824	6272	1448	Forward		cl38936| P-loop_NTPase super family	1.16 × 10^−20^
ORF7	Hypothetical protein	6344	6928	584	Forward			
ORF8	DNA primase	6921	7907	986	Forward		cl40740| DnaG super family	1.07 × 10^−9^
ORF9	Hypothetical protein	7921	8841	920	Forward			
ORF10	Putative DNA helicase	8889	10,316	1427	Forward		cl34083| SSL2 super family	1.43 × 10^−32^
ORF11	Putative DNA-binding domain protein	10,415	10,867	452	Forward		cl02600 | HTH_MerR-SF super family	1.22 × 10^−3^
ORF12	Hypothetical protein	10,874	11,257	383	Forward			
ORF13	RecA protein	11,268	12,344	1076	Forward	IPR013765	cl38936 | P-loop_NTPase super family	1.94 × 10^−50^
ORF14	Hypothetical protein	12,325	12,738	413	Forward		No hit	
ORF15	Rupredoxin-type fold protein	12,728	13,723	995	Forward		cl37788 NOB1_Zn_bind super family	8.11 × 10^−3^
ORF16	Ribonuclease-H-like domain protein	13,723	14,277	554	Forward		No hit	
ORF17	Hypothetical protein	14,386	15,360	974	Forward		No hit	
ORF18	Hypothetical protein	15,427	15,696	269	Forward		No hit	
ORF19	Hypothetical protein	15,721	16,464	743	Forward		No hit	
ORF20	Hypothetical protein	16,542	17,273	731	Forward		No hit	
ORF21	Coil containing protein	17,288	18,097	809	Forward		No hit	
ORF22	Terminase large subunit	18,084	20,057	1973	Forward		No hit	
ORF23	Hypothetical protein	20,076	20,318	242	Forward		No hit	
ORF24	Hypothetical protein	20,434	20,697	263	Forward		No hit	
ORF25	Hypothetical protein	20,739	21,005	266	Forward		No hit	
ORF26	Coil-containing protein	20,995	21,417	422	Forward		No hit	
ORF27	Pyruvate, phosphate dikinase	21,445	23,520	2075	Forward	IPR010121	cl35801 PRK09279 super family	1.21 × 10^−180^
ORF28	Hypothetical protein	23,525	24,013	488	Forward		No hit	
ORF29	Putative protein-tyrosine phosphatase	24,006	24,551	545	Forward		cl28904 PTP_DSP_cys super family	8.26 × 10^−17^
ORF30	Hypothetical protein	24,577	25,620	1043	Forward		No hit	
ORF31	Transporter	25,631	26,473	842	Forward		No hit	
ORF32	DNA polymerase I	26,473	28,830	2357	Forward	IPR002298	cl34031 PolA super family	8.32 × 10^−71^
ORF33	Portal proten	28,832	30,697	1865	Forward	IPR006944	cl19194 Phage_portal super family	2.47 × 10^−39^
ORF34	Minor head protein	30,701	31,789	1088	Forward		cl10072 Phage_Mu_F super family	1.69 × 10^−3^
ORF35	HNH endonuclease	31,789	32,304	515	Forward		pfam13392 HNH_3	5.16 × 10^−10^
ORF36	DNA methylotransferase	32,304	32,954	650	Forward	IPR007757	cl01947 MT-A70 super family	2.11 × 10^−28^
ORF37	TMhelix containing protein	33,138	33,554	416	Forward		No hit	
ORF38	Hypothetical protein	33,566	34,021	455	Forward		No hit	
ORF39	Putative DNA polymerase I	34,149	34,544	395	Forward		cl02626 DNA_pol_A super family	1.35 × 10^−5^
ORF40	Hypothetical protein	34,547	34,732	185	Forward		No hit	
ORF41	Hypothetical protein	34,755	35,123	368	Forward		No hit	
ORF42	Hypothetical protein	35,134	35,394	260	Forward		No hit	
ORF43	Hypothetical protein	35,401	35,889	488	Forward		No hit	
ORF44	Hypothetical protein	35,892	36,176	284	Forward		No hit	
ORF45	Hypothetical protein	36,182	36,481	299	Forward		No hit	
ORF46	Hypothetical protein	36,566	36,874	308	Forward		No hit	
ORF47	Hypothetical protein	36,926	38,263	1337	Forward		No hit	
ORF48	Hypothetical protein	38,309	38,902	593	Forward		No hit	
ORF49	SEC-C motif protein	38,928	39,290	362	Forward		pfam02810 SEC-C	2.55 × 10^−6^
ORF50	Hypothetical protein	39,299	39,469	170	Forward		No hit	
ORF51	putative zinc- or iron-chelating-domain-containing protein	39,466	39,927	461	Forward		No hit	
ORF52	Hypothetical protein	39,911	40,432	521	Forward		No hit	
ORF53	Hypothetical protein	40,432	40,674	242	Forward		No hit	
ORF54	Hypothetical protein	40,674	40,853	179	Forward		No hit	
ORF55	Hypothetical protein	41,325	41,765	440	Forward		No hit	
ORF56	Hypothetical protein	41,753	41,956	203	Forward		No hit	
ORF57	Hypothetical protein	41,953	42,135	182	Forward		No hit	
ORF58	Hypothetical protein	42,132	42,845	713	Forward		No hit	
ORF59	Coil containing protein	42,826	43,638	812	Forward		No hit	
ORF60	Hypothetical protein	43,644	43,952	308	Forward		No hit	
ORF61	Hypothetical protein	44,081	44,587	506	Forward		No hit	
ORF62	DUF550-domain-containing protein	44,651	45,433	782	Forward		cl04522 DUF550 super family	5.51 × 10^−7^
ORF63	Hypothetical protein	45,417	45,575	158	Forward		No hit	
ORF64	Hypothetical protein	45,683	45,877	194	Forward		No hit	
ORF65	Hypothetical protein	45,892	46,221	329	Forward		No hit	
ORF66	Hypothetical protein	46,274	46,669	395	Forward		No hit	
ORF67	Hypothetical protein	46,746	47,126	380	Forward		No hit	
ORF68	Hypothetical protein	47,178	47,639	461	Forward		No hit	
ORF69	Hypothetical protein	47,636	47,962	326	Forward		No hit	
ORF70	Hypothetical protein	47,946	48,461	515	Forward		No hit	
ORF71	Hypothetical protein	48,448	48,855	407	Forward		No hit	
ORF72	Hypothetical protein	48,861	49,031	170	Forward		No hit	
ORF73	Hypothetical protein	49,427	49,699	272	Forward		No hit	
ORF74	Hypothetical protein	49,714	49,920	206	Forward		No hit	
ORF75	Hypothetical protein	50,017	50,349	332	Forward		No hit	
ORF76	Hypothetical protein	50,408	50,743	335	Forward		No hit	
ORF77	Hypothetical protein	50,800	51,246	446	Forward		No hit	
ORF78	Hypothetical protein	51,236	51,598	362	Forward		No hit	
ORF79	Phage protein (ACLAME 851)	51,644	52,147	503	Forward		No hit	
ORF80	Hypothetical protein	52,281	52,481	200	Forward		No hit	
ORF81	VHS1018	52,523	52,774	251	Forward		No hit	
ORF82	Coil-containing protein	52,822	53,127	305	Forward		No hit	
ORF83	Hypothetical protein	53,131	53,367	236	Forward		No hit	
ORF84	Hypothetical protein	53,380	53,580	200	Forward		No hit	
ORF85	Hypothetical protein	53,580	53,723	143	Forward		No hit	
ORF86	Hypothetical protein	53,847	54,092	245	Forward		No hit	
ORF87	Hypothetical protein	54,108	54,341	233	Forward		No hit	
ORF88	Hypothetical protein	54,468	54,668	200	Forward		No hit	
ORF89	DNA Helicase	54,715	54,993	278	Forward		cl28899 DEAD-like_helicase_N super family	8.48 × 10^−3^
ORF90	Hypothetical protein	54,990	55,247	257	Forward		No hit	
ORF91	Hypothetical protein	55,244	56,659	1415	Forward		No hit	
ORF92	Hypothetical protein	56,753	57,649	896	Forward		No hit	
ORF93	Hypothetical protein	57,724	58,683	959	Forward		No hit	
ORF94	Hypothetical protein	58,814	59,128	314	Forward		No hit	
ORF95	Coil-containing protein	59,106	59,690	584	Forward		No hit	
ORF96	Hypothetical protein	59,766	60,128	362	Forward		No hit	
ORF97	Hypothetical protein	60,179	60,571	392	Forward		No hit	
ORF98	Hypothetical protein	60,568	61,002	434	Forward		No hit	
ORF99	Hypothetical protein	61,079	61,432	353	Forward		No hit	
ORF100	Hypothetical protein	61,438	61,932	494	Forward		No hit	
ORF101	Hypothetical protein	61,998	62,693	695	Forward		No hit	
ORF102	Hypothetical protein	62,697	62,978	281	Forward		No hit	
ORF103	Hypothetical protein	63,124	63,414	290	Forward		No hit	
ORF104	Hypothetical protein	63,428	63,730	302	Forward		No hit	
ORF105	Hypothetical protein	63,742	64,266	524	Forward		No hit	
ORF106	Hypothetical protein	64,266	64,538	272	Forward		No hit	
ORF107	Coil-containing protein	64,607	65,395	788	Forward		No hit	
ORF108	Hypothetical protein	65,388	67,358	1970	Forward		No hit	
ORF109	Hypothetical protein	67,424	67,774	350	Forward		No hit	
ORF110	XkdF	67,903	68,514	611	Forward		cl24270 Peptidase_S78_2 super family	2.76 × 10^−19^
ORF111	Hypothetical protein	68,517	69,686	1169	Forward		cl36772 2A1904 super family	2.50 × 10^−5^
ORF112	Major capsid protein	69,767	70,717	950	Forward	IPR024455	cl27082 Phage_capsid super family	4.87 × 10^−5^
ORF113	Coil-containing protein	70,795	71,064	269	Forward		No hit	
ORF114	Head completion adaptor	71,080	71,724	644	Forward		No hit	
ORF115	Neck protein	71,721	72,185	464	Forward		No hit	
ORF116	Tail-completion protein	72,182	72,664	482	Forward		No hit	
ORF117	Major tail protein	72,718	73,512	794	Forward	IPR016893	No hit	
ORF118	Hypothetical protein	73,601	74,035	434	Forward		No hit	
ORF119	VHS1060 protein	74,122	74,235	113	Forward		No hit	
ORF120	Tail length tape measure protein	74,241	78,374	4133	Forward		pfam10145 PhageMin_Tail	1.22 × 10^−22^
ORF121	Hypothetical protein	78,371	78,757	386	Forward		No hit	
ORF122	Hypothetical protein	78,767	79,756	989	Forward		No hit	
ORF123	Hypothetical protein	79,766	80,659	893	Forward		No hit	
ORF124	Hypothetical protein	80,662	81,960	1298	Forward		No hit	
ORF125	Hypothetical protein	81,963	82,487	524	Forward		No hit	
ORF126	Hypothetical protein	82,488	82,790	302	Forward		No hit	
ORF127	Hypothetical protein	82,787	82,960	173	Forward		No hit	

## Data Availability

The genome sequence of phage Vibrio phage Virtus is available in GenBank under accession number OK381870. The associated BioProject and BioSample accession numbers are PRJNA764828 and SAMN21529761, respectively.
